# Metagenomic next-generation sequencing of cell-free and whole-cell DNA in diagnosing central nervous system infections

**DOI:** 10.3389/fcimb.2022.951703

**Published:** 2022-09-27

**Authors:** Lili Yu, Ye Zhang, Jiemin Zhou, Yu Zhang, Xuejiao Qi, Kaixuan Bai, Zheng Lou, Yi Li, Han Xia, Hui Bu

**Affiliations:** ^1^ Department of Neurology, The Second Hospital of Hebei Medical University, Shijiazhuang, China; ^2^ Department of Scientific Affairs, Hugobiotech Co., Ltd., Beijing, China

**Keywords:** CNS infection, critically ill patients, sensitivity, mNGS, cell-free DNA, whole-cell DNA

## Abstract

**Background:**

Central nervous system (CNS) infections pose a fatal risk to patients. However, the limited sample volumes of cerebrospinal fluid (CSF) and low detection efficiency seriously hinder the accurate detection of pathogens using conventional methods.

**Methods:**

We evaluated the performance of metagenomics next-generation sequencing (mNGS) in diagnosing CNS infections. CSF samples from 390 patients clinically diagnosed with CNS infections were used for the mNGS of cell-free DNA (cfDNA) (*n* =394) and whole-cell DNA (wcDNA) (*n* =150).

**Results:**

The sensitivity of mNGS using cfDNA was 60.2% (237/394, 95% confidence interval [CI] 55.1%–65.0%), higher than that of mNGS using wcDNA (32.0%, 95% [CI] 24.8%–40.2%, 48/150) and conventional methods (20.9%, 95% [CI] 16.2%–26.5%, 54/258) (*P* < 0.01, respectively). The accuracy of mNGS using cfDNA in positive samples was 82.6%. Most of viral (72.6%) and mycobacterial (68.8%) pathogens were only detected by the mNGS of cfDNA. Meningitis and encephalitis with *Streptococcus pneumoniae* infection might be more likely to result in critically ill diseases, while Human alphaherpesvirus 3 was prone to cause non-critically ill diseases.

**Conclusions:**

This is the first report on evaluating and emphasizing the importance of mNGS using CSF cfDNA in diagnosing CNS infections, and its extensive application in diagnosing CNS infections could be expected, especially for viral and mycobacterial CNS infections.

## Introduction

Central nervous system (CNS) infections are mainly caused by bacteria, fungi, viruses, and parasites (Zhang et al., 2020). They can result in meningitis, encephalitis, abscess, and so on (Zhang et al., 2020) and lead to 100% mortality in some cases (Ma et al., 2015). Meningitis affected more than 1.2 million people each year (Cain et al., 2019) with a mortality of more than 26% (320,000 people died from meningitis) in 2016 (Cain et al., 2019; Zhang et al., 2020). Of the deaths from meningitis, approximately 40% were due to bacterial meningitis (approximately 120,000 deaths) (Cain et al., 2019; Zhang et al., 2020). Viral encephalitis and meningitis contributed to 34% of adult CNS infections, and 14% of meningitis was tuberculous meningitis (TBM) (Ho Dang Trung et al., 2012). However, it is difficult to distinguish TBM from cryptococcal meningitis (CM) and viral meningitis due to the lack of specificity in clinical presentation and cerebrospinal fluid (CSF) parameters of those patients (Abassi et al., 2015; Leonard, 2017; Ji et al., 2020). In addition to death, some unoptimistic prognoses may be generated if timely diagnosis and proper therapy were not provided, including neurological disability (Berhane et al., 2021). Accordingly, not only timely but also accurate diagnosis are needed to improve prognoses and decrease mortality.

The conventional CSF culture method, as the most commonly used diagnostic tool, can only identify approximately 30%–40% of CNS infections (Leber et al., 2016) whose detection rates are as low as 6% in developing countries (Zhang et al., 2020), while some pathogens take weeks to grow (Gu et al., 2021). Although hypothesis-based PCR (Gu et al., 2021), Xpert (Bahr et al., 2018), and BioFire FilmArray Panel (Leber et al., 2016) are more accurate and sensitivities for some pathogens can reach 100% (Leber et al., 2016), they either need a prior hypothesis of the causative pathogens (Gu et al., 2021) or are restricted to the detection of a limited number of pathogens (Zhang et al., 2020). Unbiased metagenomics next-generation sequencing (mNGS) has been extensively used in clinical infections (Wilson et al., 2019; Chen et al., 2021a; Gu et al., 2021; Chen et al., 2021b) after the first successful application in diagnosing CNS infections (Wilson et al., 2014). Most importantly, the mNGS of cell-free DNA (cfDNA) has been proven to be a promising tool for detecting and identifying pathogens in body fluids (Gu et al., 2021).

However, long-held conflict in the location of proliferation and infection between intracellular and extracellular pathogens (Casadevall and Fang, 2020) challenged whether the mNGS of cfDNA can accurately detect causative pathogens from CSF. Compared to the mNGS of cfDNA, although the mNGS of whole-cell DNA (wcDNA) obtained from the differential lysis method can detect more reads per million (RPM) of *Cryptococcus neoformans*, its detection of trace *Mycobacterium tuberculosis* was hindered (Ji et al., 2020). The differential lysis method can significantly improve the detection performance of dominant pathogens at the expense of trace pathogens (Thoendel et al., 2018; Gu et al., 2021). Furthermore, the detection rate (Zhang et al., 2020) of mNGS directly using the wcDNA of CSF samples without differential lysis was only approximately 50%. We hypothesized that the performance of mNGS using cfDNA is better than that of mNGS using wcDNA in CNS infections.

To verify our hypothesis, we retrospectively enrolled patients with CNS infections mainly diagnosed as bacterial, viral, mycobacterial, or cryptococcal infections by conventional methods. The remaining CSF samples from those patients were used to evaluate the accuracies of mNGS using cfDNA and wcDNA, including the positive rate and sensitivity, while the performance of the conventional methods and pathogen profiles in CNS infections were also summarized.

## Materials and methods

### Ethics statement

Study protocols were approved by the Ethical Review Committee of the Second Hospital of Hebei Medical University, Hebei, China (approval no. 2020-P027). All procedures were in accordance with the ethical standards of the responsible committee on human experimentation (institutional and national) and with the Helsinki Declaration of 1975, as revised in 2000. Informed consent was obtained from all patients enrolled in the study or their next kin/guardian.

### Sample selection and patient division

For this retrospective study, a total of 478 consecutive patients admitted to the Second Hospital of Hebei Medical University of China from June 2018 to June 2021 and diagnosed as CNS infections were assessed for eligibility. The definition of suspected CNS infections was based on the diagnostic criteria of encephalitis, meningitis, meningoencephalitis, and meningomyelitis of a previous study (Schibler et al., 2019): a) patients with no alternative cause altered the mental status (decreased or altered level of consciousness, lethargy, or personality change) for ≥24 h and with at least two following symptoms, including fever ≥38.2°C within 72 h before or after admission, generalized or partial seizures not fully attributable to a preexisting seizure disorder, new onset of focal neurologic findings, CSF leukocyte count ≥5 M/L, abnormality of brain parenchyma on neuroimaging indicating encephalitis, and abnormality by electroencephalography consistent with encephalitis; b) patients with CSF leukocyte count ≥5 M/L and with at least two following symptoms, including headache, fever (≥38.2°C), photo- and/or phonophobia, and neck stiffness; and c) patients with asymmetrical flaccid weakness combined with reduced/absent reflexes and sensory symptoms or with the hyperintensities of the spinal cord on T2-weighted magnetic resonance imaging.

For all patients with symptoms meeting the above definition, the clinical information, including epidemiology, clinical manifestations, laboratory test results, imaging results, conventional diagnostic results, outcomes after anti-infective treatments, and 2-month follow-ups, was assessed for the final diagnosis by two clinicians independently. Any discrepancies in the determination of etiology were resolved by direct communication with treating physicians or by a mutual consensus with a third clinician. Only patients with confirmed CNS infections were assessed for eligibility in this study.

The remaining CSF samples used for further mNGS tests from patients with CNS infections were screened using the following exclusion criteria: 1) the volume of CSF was not enough for mNGS detection; 2) the quality of CSF was too low to perform mNGS, such as hemolytic CSF; 3) the constructed DNA libraries of samples were unqualified and were not next sequenced; 4) final diagnoses were indefinite; and 5) the patients declined to participate. Given the low incidence rates of CNS infections and the limitation in funding, we retrospectively enrolled more patients using the limited funding without formal statistical considerations.

Clinical diagnostic tests included routine bacterial and fungal smears and cultures, India Ink, Alcian blue staining, acid-fast staining, serum antigen and antibody tests, interferon-γ release assays, tubercalin test, and PCR. The definition of encephalitis, meningitis, and meningoencephalitis was based on a previous study (Schibler et al., 2019). According to the modified Rankin Scale (mRS) (Sulter et al., 1999) and intensive care unit (ICU) admission, we divided patients into critically or non-critically ill patients.

The remaining 394 CSF samples from these 390 patients were transported to Hugobiotech (Hugobiotech, Beijing, China) on dry ice for mNGS tests. During the process from the wet lab to dry lab of mNGS, corresponding researchers were not informed with any clinical results, including laboratory tests, clinical information, and the final clinical diagnosis.

### DNA extraction

CfDNA and wcDNA were extracted from CSF (~2 ml) using the QIAamp DNA Micro Kit (QIAGEN, Hilden, Germany) based on its manual. For cfDNA extraction, the cells in samples were removed by centrifugation and the supernatant was collected for the subsequent extraction, while the total CSF sample was directly used for wcDNA extraction without centrifugation. In this study, the extraction of cfDNA and wcDNA was performed in 394 and 150 samples, respectively. The simple random sampling method was used for the random selection of samples for wcDNA extraction.

### Metagenomics next-generation sequencing detection

DNA libraries were constructed using the QIAseq™ Ultralow Input Library Kit (QIAGEN, Hilden, Germany) according to the instructions. The quality of constructed libraries was assessed by Qubit (Thermo Fisher, Waltham, USA) and Agilent 2100 Bioanalyzer (Agilent Technologies, Palo Alto, CA, USA). All qualified libraries were sequenced on the Nextseq 550 platform (Illumina, San Diego, CA, USA), with 75 bp single-end reads of approximately 20 million per library after sequencing. Adapters and short (<35 bp), low-quality (Q < 30), and low-complexity reads were removed to generate clean data using bowtie2, which were then mapped to the human reference database (hg38) to filter out human host DNA reads. The remaining reads were finally blasted against Microbial Genome Databases (http://ftp.ncbi.nlm.nih.gov/genomes/) using Burrows–Wheeler Aligner software, followed by species annotation analysis using the least common ancestor method. Negative controls (sterile deionized water) and positive controls (synthesized fragments with known quantities) were established for each batch of experiments using the same wet lab procedures and bioinformatics analysis as the clinical samples. The read number and RPM of each detected microbe were calculated. For detected microbes, including bacteria (*Mycobacterium* excluded), fungi (*Cryptococcus* excluded), and parasites, a positive mNGS result was given when its coverage ranked in the top 10 of similar microbial species (or genera) and was absent in the negative control (“No template” control, NTC) or when its ratio of RPM between the sample and NTC (RPM_sample_/RPM_NTC_) > 10 if RPM_NTC_≠0. For viruses, *Mycobacterium*, and *Cryptococcus*, a positive mNGS result was considered when at least one unique read was mapped to the species level and absent in NTC or RPM_sample_/RPM_NTC_ > 5 when RPM_NTC_≠0.

### Statistical analysis

Descriptive statistics were used for demographic information as mean ± SD. The proportion of the specified group was presented as percentage. The positive rate and sensitivity of mNGS using cfDNA, mNGS using wcDNA, and conventional methods were calculated (percentage with 95% confidence interval [CI]) and compared (chi-square test). According to the clinical manifestations, the patients were also divided into the critically ill group and non-critically ill group, as well as the meningitis group and encephalitis group, respectively. The pathogen spectrum characteristics between different groups were compared (chi-square test). A *p*-value less than 0.05 was considered statistically significant.

### Data availability

Sequencing data were deposited to the National Genomics Data Center (http://ngdc.cncb.ac.cn) under accession number PRJCA008673. The authors declare that the main data supporting the findings are available within this article. The other data generated and analyzed for this study are available from the corresponding author upon reasonable request.

## Results

### Patient baseline

A total of 478 consecutive patients diagnosed as CNS infections from June 2018 to June 2021 were assessed for eligibility, while, combined with exclusion criteria for samples, 394 CSF samples from 390 patients were enrolled for this study. There were 256 men and 134 women (**Table 1**). The average age was 43.6 ± 1.7 years (from 13 to 85 years old). The average length of hospital stay was 19.9 ± 1.2 days (from 2 to 96 days). A total of 100 patients (25.6%) experienced underlying diseases, including diabetes, hypertension, hepatitis B, the postoperation of diseases, skull defect, and sinusitis. A total of 38 patients (9.7%) had immunodeficiency, including systemic lupus erythematosus, nephrotic syndrome, postoperation of cancer, and HIV. Furthermore, 35 patients had other infections, such as pneumonia. The common symptoms of the 390 patients with CNS infections were fever (288), headache (277), nausea and vomiting (142), meningeal irritation (134), consciousness disorder (115), focal neurological deficit (83), epilepsy (74), and behavior change (38). The most common diseases caused by CNS infections were meningitis (168) and encephalitis (169), followed by meningoencephalitis (41), meningomyelitis (9), and brain abscess (3). The 109 patients (27.95%) with an MRS score of ≥4 admitted to ICU for treatment were defined as critically ill patients.

**Table 1:** The baseline of the patients enrolled in this study.

	Number of patients
**Male (%)**	256 (66.3%)
**Age (year)**	43.6 ± 1.7
**Hospital stay (day)**	19.9 ± 1.2
**Underlying diseases**	100
**Immunosuppressed diseases**	38
**With critical illnesses**	109
**CNS infectious diseases**	
Meningitis	168
Encephalitis	169
Meningoencephalitis	41
Encephalomyelitis	9
Brain abscess	3
Coinfections of other organs	35
**Symptoms**	
Fever	288
Headache	277
Nausea/vomiting	142
Meningeal irritation	134
Consciousness disorder	115
Focal neurological deficit	83
Epilepsy	74
Behavior change	38

### Performance of metagenomics next-generation sequencing using cell-free DNA and whole-cell DNA

**Figure 1:**
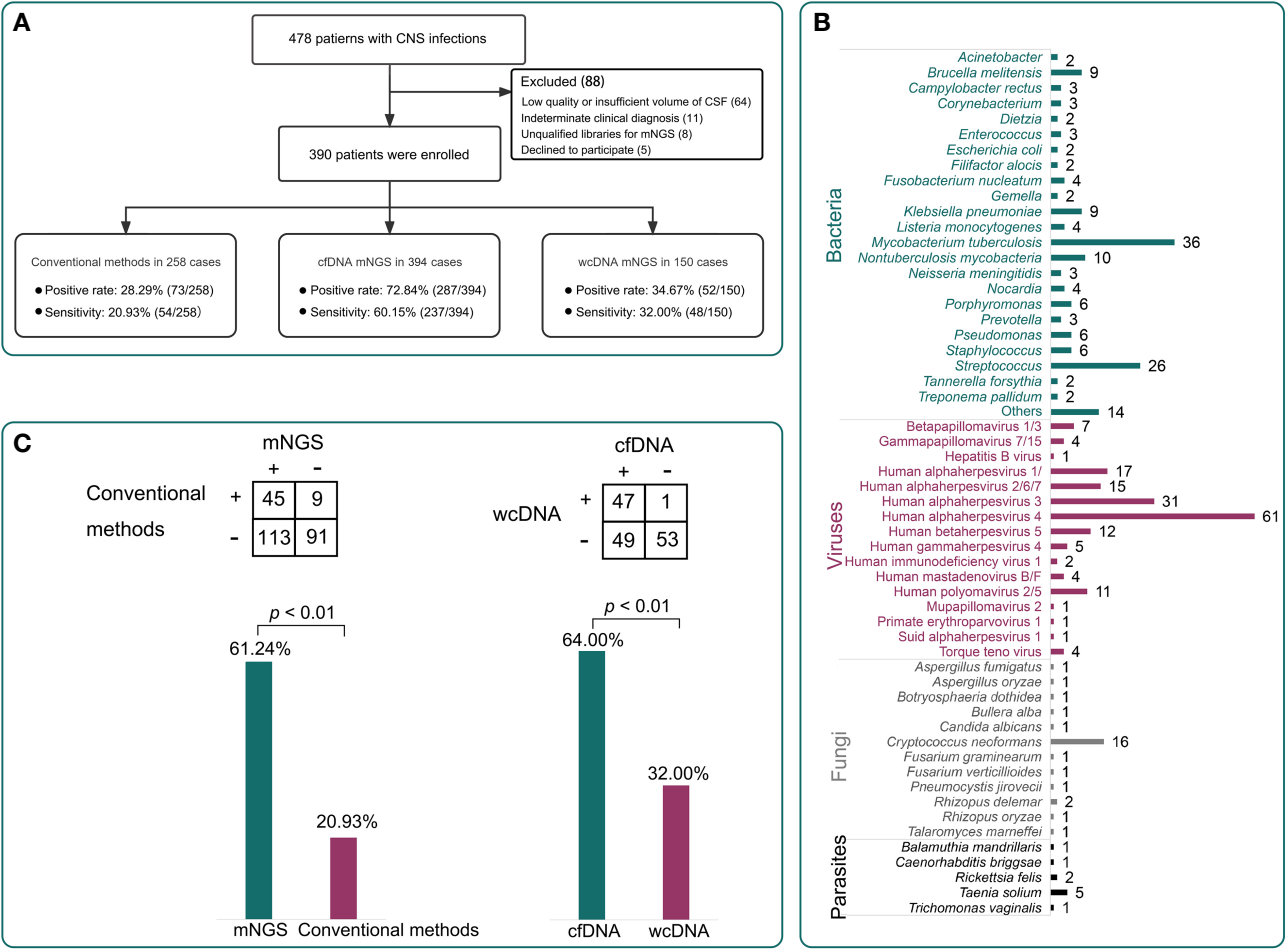
Patient enrollment and the performance of different methods. **(A)**, A total of 390 patients were enrolled in this study. The positive rates of conventional methods, metagenomics next-generation sequencing (mNGS) using cell-free DNA (cfDNA), and mNGS using whole-cell DNA (wcDNA) were 28.3%, 72.8%, and 34.7%, respectively. The sensitivities were 20.9%, 60.2%, and 32.0%, respectively. **(B)**, The pathogen profiles by the mNGS of cfDNA. **(C)**, Comparisons among different methods. The mNGS of cfDNA showed significantly higher sensitivity than the mNGS of wcDNA and conventional methods (*P* < 0.01).

We randomly selected 150 CSF samples from the enrolled 390 patients to perform mNGS tests using both cfDNA and wcDNA simultaneously (**Figure 1A**). The positive rate of mNGS using cfDNA (66.7%, 95% [CI] 58.5%–74.0%, 100/150) was higher than that of mNGS using wcDNA (34.7%, 95% [CI] 27.2%–42.9%, 52/150) (*P*<0.01). The sensitivity of mNGS using cfDNA (64.0%, 95% [CI] 55.71%–71.55%, 96/150) was also higher than that of mNGS using wcDNA (32.0%, 95% [CI] 24.8%–40.2%, 48/150) (*P*<0.001) (**Figure 1C**). Furthermore, among those samples with mNGS results coincident with the final clinical diagnoses, only 1 out of 48 samples by the mNGS of wcDNA cannot be confirmed by the mNGS of cfDNA, while the mNGS of wcDNA did not identify causative pathogens from 51% of samples (49/96) successfully diagnosed by the mNGS of cfDNA. Interestingly, approximately 98% of the 49 samples were viral (*n* = 36) and mycobacterial infections (*n* = 11). Given the slight differences in mNGS detection between viral and mycobacterial infections, we further evaluated the performance of mNGS using both cfDNA and wcDNA at the pathogen level.

**Figure 2:**
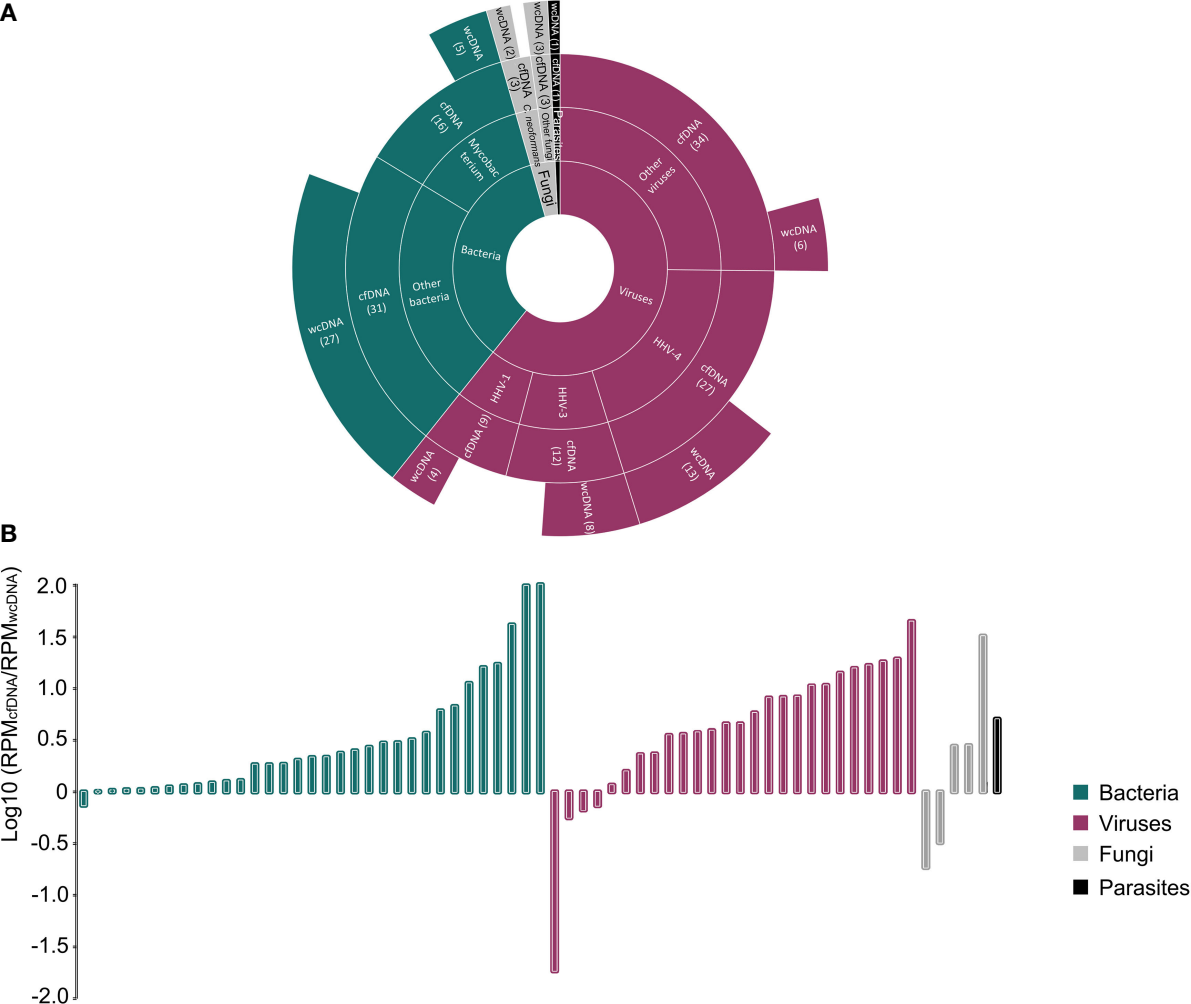
Comparison between the mNGS of cfDNA and wcDNA. **(A)**, Summaries of case numbers by detected pathogens, including bacteria, viruses, fungi, and parasites. The mNGS of cfDNA detected more viral and mycobacterial infections than the mNGS of wcDNA. **(B)**, Difference in the number of detected RPMs for the same pathogen between the mNGS of cfDNA and wcDNA. The value of >0 represents that the number of detected RPMs by the mNGS of cfDNA is higher than that by the mNGS of wcDNA.

We found a significant difference in the detection of viruses and *Mycobacterium* (**Figure 2A**). In general, the number of detected RPMs using the mNGS of cfDNA was significantly higher than that using the mNGS of wcDNA (94,344 vs. 27,153, *P*<0.05) (**Figure 2B**). For *Mycobacterium* detection, only 5 positive results were found by the mNGS of both cfDNA and wcDNA, while 11 positive results were only detected by the mNGS of cfDNA. Furthermore, a higher performance of mNGS using cfDNA than that of mNGS using wcDNA can also be found in detecting viruses (82 vs. 31), including Human gammaherpesvirus 4 (EBV, 27 vs. 13), Human alphaherpesvirus 3 (VZV, 12 vs. 8), and Human alphaherpesvirus 1 (HSV, 9 vs. 4). The above results unravel that the accuracy of mNGS using CSF cfDNA is higher than that of mNGS using CSF wcDNA in diagnosing CNS infections, especially for viral and mycobacterial CNS infections.

### Performance of metagenomics next-generation sequencing using cell-free DNA and conventional methods

**Figure 3:**
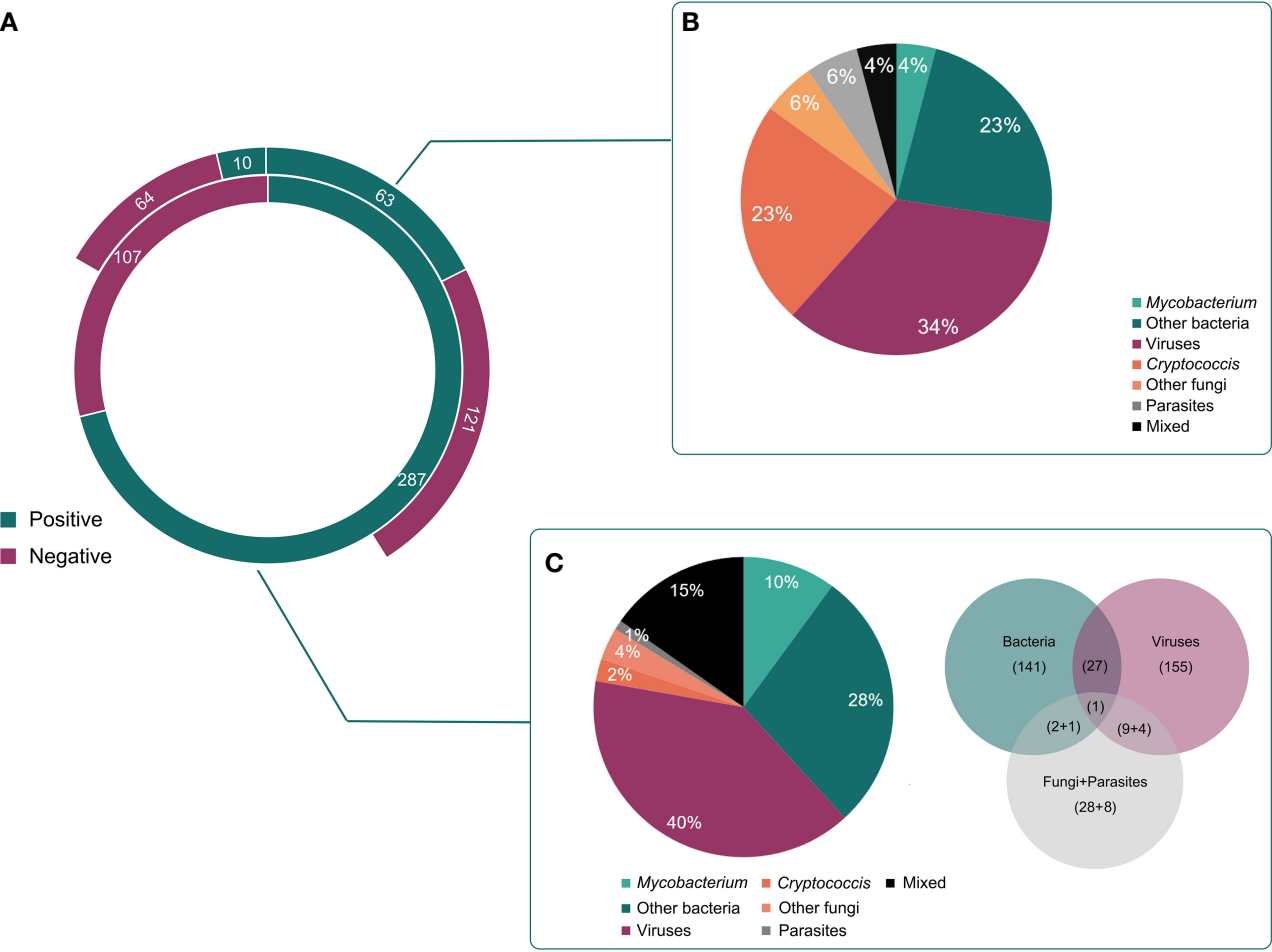
Different kinds of infections by mNGS and conventional methods. **(A)**, The detected results of mNGS and conventional methods in all enrolled patients. **(B)**, Different kinds of infections in positive cases detected by conventional methods. **(C)**, Different kinds of infections in positive cases detected by mNGS.

mNGS using cfDNA was performed in other CSF samples to assess its importance in diagnosing CNS infections, while conventional methods were previously conducted on 258 CSF samples to assist diagnosis (**Figure 3A**). Taking the final clinical diagnosis as a gold standard, the performance of mNGS using cfDNA was much better than that of conventional methods (**Figure 1A**). mNGS and conventional methods respectively detected causative pathogens from 287 (72.8%) out of 394 samples and 73 (28.3%) out of 258 samples. Furthermore, the sensitivity of mNGS (60.2%, 95% [CI] 55.1%–65.0%, 237/394) was significantly higher than that of conventional methods (20.9%, 95% [CI] 16.2%–26.5%, 54/258) (*P*<0.01). Among the 258 samples, mNGS can detect causative pathogens from 113 samples with negative results by conventional methods. The above results unravel that mNGS detection using CSF cfDNA should be considered as a preferred examination in diagnosing CNS infections.

### Pathogen profiles detected by conventional methods and mNGS using cfDNA

Pathogens detected by conventional methods were limited to some species. The most commonly detected causative pathogens were *Cryptococcus* (*n* = 17), HSV (*n* = 15), Human betaherpesvirus 5 (CMV) (*n* = 18), *Klebsiella pneumoniae* (*n* = 4), *Brucella melitensis* (*n* = 4), and *M. tuberculosis* (*n* = 3) (**Figure 3B**). Conversely, mNGS identified a total of 65 bacteria, 23 viruses, 12 fungi, and 5 parasites (**Figure 1B**). Capturing more causative pathogens can provide a comprehensive reference for accurate infection diagnosis.

Based on mNGS detection, we further classified the patients into bacterial infection (*n* = 141), viral infection (*n* = 155), fungal infection (*n* = 28), and parasitic infection (*n*=9), while 44 patients were coinfected with different kinds of pathogens (**Figure 3C**). The most common bacterial causative pathogens were *M. tuberculosis* (*n* = 36), *Streptococcus pneumoniae* (*n* = 15), non-tuberculous mycobacteria (*n* = 10), *B. melitensis* (*n* = 9), and *K. pneumoniae* (*n* = 9). *C. neoformans* (*n* = 16) was the most common fungal causative pathogen, which was similar to the result of conventional methods. In addition, mNGS results showed that EBV (*n* = 61) contributed approximately 40% to the viral infection, which was only detected in three samples using conventional methods.

### Performance of metagenomics next-generation sequencing using cell-free DNA in infection diagnosis of critically ill patients

Compared with non-critically ill patients, a better performance of mNGS in infection diagnosis was found for critically ill patients. After division, 110 and 284 CSF samples were respectively obtained from critical and non-critical ill patients. Positive results were found in 88 samples of critically ill patients and 199 samples of non-critically ill patients. Taking the final diagnoses as a gold standard, a high sensitivity of mNGS was found in both critically (65.5%, 95% [CI] 55.7%–74.1%, 72/110) and non-critically (58.1%, 95% [CI] 52.1%–63.9%, 165/284) ill patients. Our findings show that the application of mNGS using CSF cfDNA is beneficial to accurately identify the causative pathogens of CNS infections.

**Figure 4:**
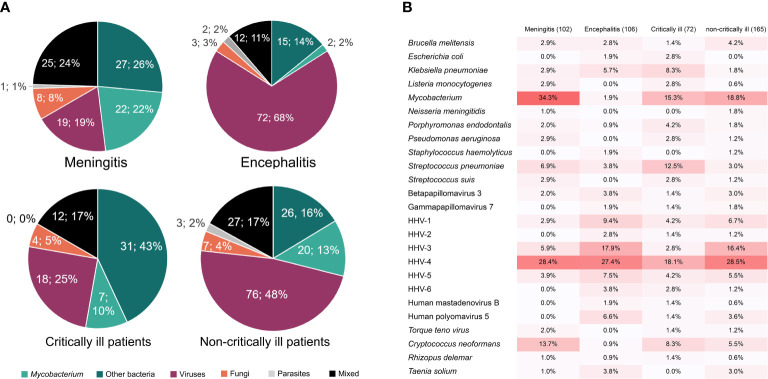
The percentages of causative pathogens in meningitis, encephalitis, critically ill, and non-critically ill patients by mNGS using cfDNA. **(A)**, Different kinds of infections in meningitis, encephalitis, critically ill, and non-critically ill patients. **(B)**, Main causative pathogens in meningitis, encephalitis, critically ill, and non-critically ill patients.

Furthermore, we found the difference in causative pathogens between critically and non-critically ill patients (**Figure 4**). More than 52% of critically ill patients (38/72) were attributed to bacterial infections, while the percentage of viral infections was only 25% (18/72). However, non-critically ill patients were much more susceptible to viral infection (49.1%, 81/165) rather than bacterial infection (28.5%, 47/165) (**Figure 4A**). The analysis of pathogen profiles (**Figure 4B**) showed that *M. tuberculosis* and EBV were commonly detected in both critically and non-critically ill patients, while high ratios of *S. pneumoniae* and VZV were respectively found in critically and non-critically ill patients. High ratios of bacterial (~58%) and viral (83%) infections were also found in meningitis and encephalitis patients, respectively. However, the ratios of meningitis were similar to those of encephalitis in both critically and non-critically ill patients. The above raises a question of which types of infection in meningitis or encephalitis are more likely to result in critically ill diseases.

### Causative pathogens of meningitis and encephalitis detected by modified next-generation sequencing using cell-free DNA

Different infection types were found in meningitis and encephalitis patients. Causative pathogens in 102 meningitis and 106 encephalitis patients were further confirmed by mNGS (**Figure 4**). We found that *Mycobacterium* was the dominant pathogen in meningitis patients (34.3%), which was detected in few encephalitis patients (1.9%). Combined with non-differential distribution between critically and non-critically ill patients, we proposed that *Mycobacterium* is more likely to cause meningitis. The low ratios of *S. pneumoniae* in both meningitis and encephalitis patients and high ratios in critically ill patients indicate that meningitis and encephalitis with *S. pneumoniae* infection might be more likely to result in critically ill diseases, whereas the high ratios of VZV in both encephalitis patients (17.9%) and non-critically ill patients (16.4%) suggest that CNS VZV infection might only result in non-critically ill diseases.

## Discussion

This is the first report on evaluating the accuracies of mNGS using CSF cfDNA and CSF wcDNA and on emphasizing the advantage of mNGS using cfDNA in diagnosing CNS infections, especially for viral and mycobacterial CNS infections. The sensitivity of mNGS using cfDNA can reach up to 60.2%, with an accuracy of 82.6% among the patients with positive results. The pathogen profiles of mNGS using cfNDA were analyzed. *M. tuberculosis* and EBV were commonly detected in both critically and non-critically ill patients. Meningitis and encephalitis with *S. pneumoniae* infection might be more likely to result in critically ill diseases, while CNS infection with VZV might only result in non-critically ill diseases.

The mNGS of CSF cfDNA exhibited better performance in diagnosing CNS infections than the mNGS of wcDNA and conventional methods. The limited volumes of CSF samples (Ji et al., 2020) and low detection efficiency (Leber et al., 2016; Bahr et al., 2018; Ge et al., 2021) of conventional methods hindered the accurate detection of pathogens, while mNGS can achieve an unbiased detection of pathogens from different kinds of samples (Gu et al., 2021). The sensitivity of mNGS was determined by the pathogen DNA ratio in a sample (Ebinger et al., 2021). In our study, during wcDNA extraction, the differential lysis method was not included to filter human DNA from the CSF sample (Thoendel et al., 2018), while cfDNA was directly extracted from the low-cellularity supernatant of the CSF sample (Ji et al., 2020; Gu et al., 2021), resulting in the fact that the pathogen DNA ratio of cfDNA might be higher than that of wcDNA for the same CSF sample. This may be the reason why the sensitivity of mNGS using cfDNA is higher than that of mNGS using wcDNA not only in our study but also in the published study on CNS infection (Wilson et al., 2019). Accordingly, the detection of mNGS using CSF cfDNA should be considered as a preferred examination in diagnosing CNS infections.

Pathogens causing viral and mycobacterial CNS infections were susceptible to mNGS detection using cfDNA rather than using wcDNA. Given the low loads of viruses (Carbo et al., 2020) and *Mycobacterium* (Brown et al., 2016) in CSF, a better performance of mNGS using cfDNA in our study may be attributed to its advantage in DNA extraction and bioinformatics analysis for trace pathogens. First of all, wcDNA extraction involves the cell wall lysis of pathogens using an extraction kit (Wilson et al., 2019), increasing the risk of DNA degradation (Thoendel et al., 2018; Gu et al., 2021) and influencing the DNA recovery rate of pathogens with low loads. Furthermore, the DNA of intracellular pathogens can exist in body fluids in the form of cfDNA (Casadevall and Fang, 2020), and we have proven that the mNGS of cfDNA can successfully detect *Mycobacterium* and viruses from bronchoalveolar fluid samples (Chen et al., 2021a).

Furthermore, the difference in the pathogen DNA ratio can affect the denoising performance of the bioinformatics algorithm in the dry-lab pipeline of mNGS, influencing the subsequent identification of trace pathogens (Ji et al., 2020). We previously found that the bioinformatics algorithm used in this study can decode and identify two reads of a desired pathogen (Wu et al., 2020). A lower DNA recovery rate and worse denoising performance comprehensively reduce the accuracy of mNGS using wcDNA in detecting trace pathogens. Accordingly, the mNGS of cfDNA is much more suitable for diagnosing viral and mycobacterial CNS infections than the mNGS of wcDNA.

A high percentage of mycobacterial CNS infection was found in critically ill patients. In our study, most of the mycobacterial infections were meningitis (**Figure 4**). Mycobacterial meningitis can cause high morbidity (25%) and mortality (15%–40%) (Komorowski et al., 2021), whose manifestation is similar to bacterial or viral meningitis (Sethi and Davies, 2020). Furthermore, mycobacterial meningitis is subacute (Sethi and Davies, 2020; Komorowski et al., 2021) and the detection rates by conventional methods are low (Wang et al., 2019), resulting in delayed diagnosis and treatment. Accordingly, the use of conventional methods without mNGS for diagnosing mycobacterial meningitis may subsequently lead to infection progression, increasing the percentage of mycobacterial infection in critically ill patients. Based on our findings, an extensive application of mNGS using CSF cfDNA in diagnosing mycobacterial CNS infection could be expected.

mNGS using cfDNA can quickly and accurately unveil the prevalence of viral CNS infections and define their roles in causing critical and non-critical illnesses, especially for EBV and VZV. EBV is responsible for 2%–5% of viral encephalitis and meningitis patients, and a review covering a 10-year period revealed that EBV was occasionally detected in CSF using PCR (Lee et al., 2021), which was consistent with our result of conventional methods (2/258). However, we detected EBV in 61 out of 394 samples using cfDNA mNGS. Latent infection in B lymphocytes is essential for the persistence and transmission of EBV, which is estimated to infect >90% of adults worldwide (Hong et al., 2021). Latency (Hong et al., 2021) and low load (Carbo et al., 2020) might allow EBV to escape from detection by PCR, further indicating that the limit of detection for mNGS is much lower than that for PCR. Furthermore, the high percentages of EBV in both critically and non-critically ill patients emphasize that more clinical attention should be paid to latent viral infection.

We found a high ratio of VZV in non-critically ill patients (median age: 48.5 years old), which was consistent with the long-held opinion that VZV preferentially affected elder patients (Mailles et al., 2009; Grahn and Studahl, 2015). With age, immunosenescence and treatment-related immunosuppression provide conditions for the reactivation and infection of latent VZV (Arruti et al., 2017). Theoretically, elder critically ill patients (median age: 63 years old) were much more susceptible to VZV infection than non-critically ill patients. However, VZV was only detected in three critically ill patients (37, 46, and 69 years old) using mNGS. The continuous use of antimicrobial drugs can significantly influence the pathogen detection of CNS infection using mNGS (Zhang et al., 2020). VZV might be sensitive to specific therapy for critically ill patients in ICU, leading to a low detection rate in critically ill patients. The above suggests that the timing for mNGS in CNS infection needs to be dug out to accurately identify pathogens.

## Limitations

This is a single-center retrospective study; potential bias from participant recruitment and data collection cannot be ruled out, and more samples from multiple hospitals are needed to further evaluate the performance of mNGS, especially for the comparison between the mNGS of cfDNA and wcDNA. Negative controls from healthy cases and those with non-infectious CNS diseases should be included to detect the potential of mNGS in ruling out infection. In addition, the potential of mNGS to help with the timely adjustment of treatments should also be evaluated. The pathogenesis of viral CNS infections should be further explored.

## Conclusions

Our findings highlight the importance of mNGS using CSF cfDNA in diagnosing CNS infections, especially for viral and mycobacterial CNS infections. The most commonly detected pathogens by mNGS were *M. tuberculosis* and EBV. Furthermore, more clinical attention should be paid to CNS infections with *S. pneumoniae* and latent viruses. Based on our findings, an extensive application of mNGS using CSF cfDNA in diagnosing CNS infection could be expected.

## Data availability statement

The datasets presented in this study can be found in online repositories. The names of the repository/repositories and accession number(s) can be found below: https://ngdc.cncb.ac.cn/, PRJCA008673.

## Ethics statement

Study protocols were approved by the Ethical Review Committee of the Second Hospital of Hebei Medical University, Hebei, China (approval no. 2020-P027). All procedures were in accordance with the ethical standards of the responsible committee on human experimentation (institutional and national) and with the Helsinki Declaration of 1975, as revised in 2000. Informed consent was obtained from all patients enrolled in the study or their next kin/guardian.

## Author contributions

ZL and HB designed the paper. LY, YeZ, and JZ drafted the manuscript. LY, YuZ, XQ, KB, and YL carried out the clinical care and management of the patients. HX performed the mNGS tests and analyzed the data. YeZ and JZ revised the manuscript. All authors approved the final manuscript as submitted and agreed to be accountable for all aspects of the work.

## Funding

This work was supported by the Science and Technology Program of Hebei (No.20377790D) and the Science and Technology Project of Xi’an (No. 21RGSF0013).

## Conflict of interest

YeZ, JZ, ZL, and HX are employed by Hugobiotech Co., Ltd.

The remaining authors declare that the research was conducted in the absence of any commercial or financial relationships that could be construed as a potential conflict of interest.

## Publisher’s note

All claims expressed in this article are solely those of the authors and do not necessarily represent those of their affiliated organizations, or those of the publisher, the editors and the reviewers. Any product that may be evaluated in this article, or claim that may be made by its manufacturer, is not guaranteed or endorsed by the publisher.
